# Supplementary material for: Heteroalleles in Common Wheat: Multiple Differences between Allelic Variants of the Gli-B1 Locus
Source: Int J Mol Sci. 2021 Feb 12;22(4):1832. doi: 10.3390/ijms22041832 (PMC7917834; doi:10.3390/ijms22041832)
Supplement: Supplementary file 1 [file ijms-22-01832-s001.pdf]

SUPPLEMENTARY MATERIAL

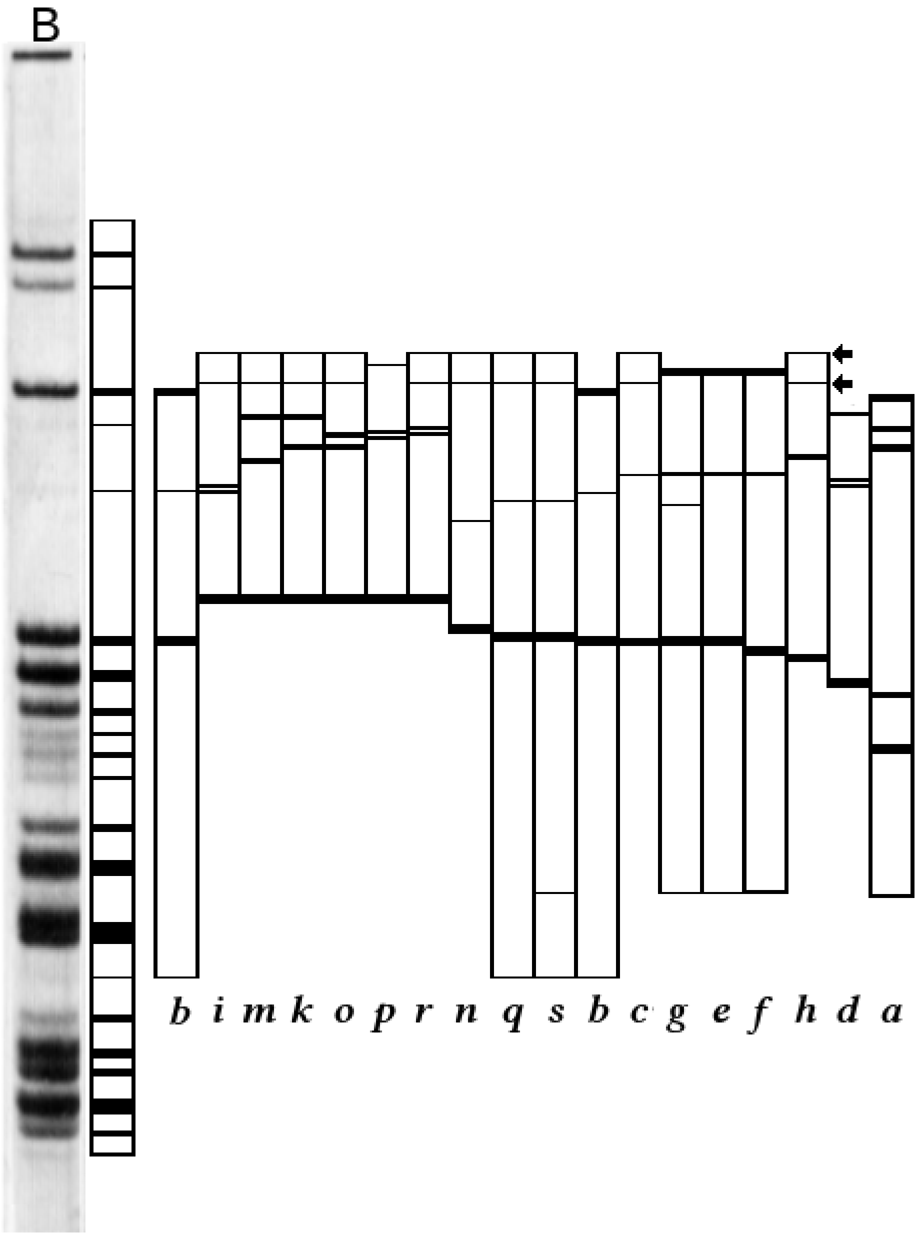

Figure S1

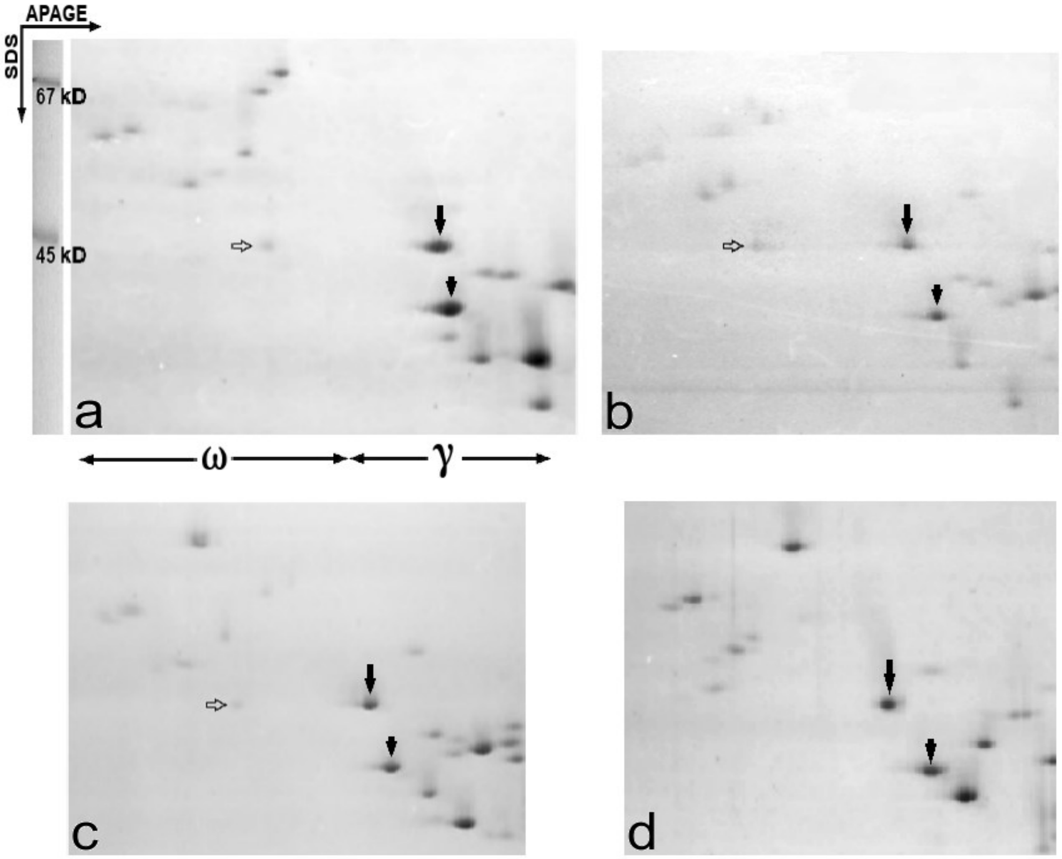

Figure S2

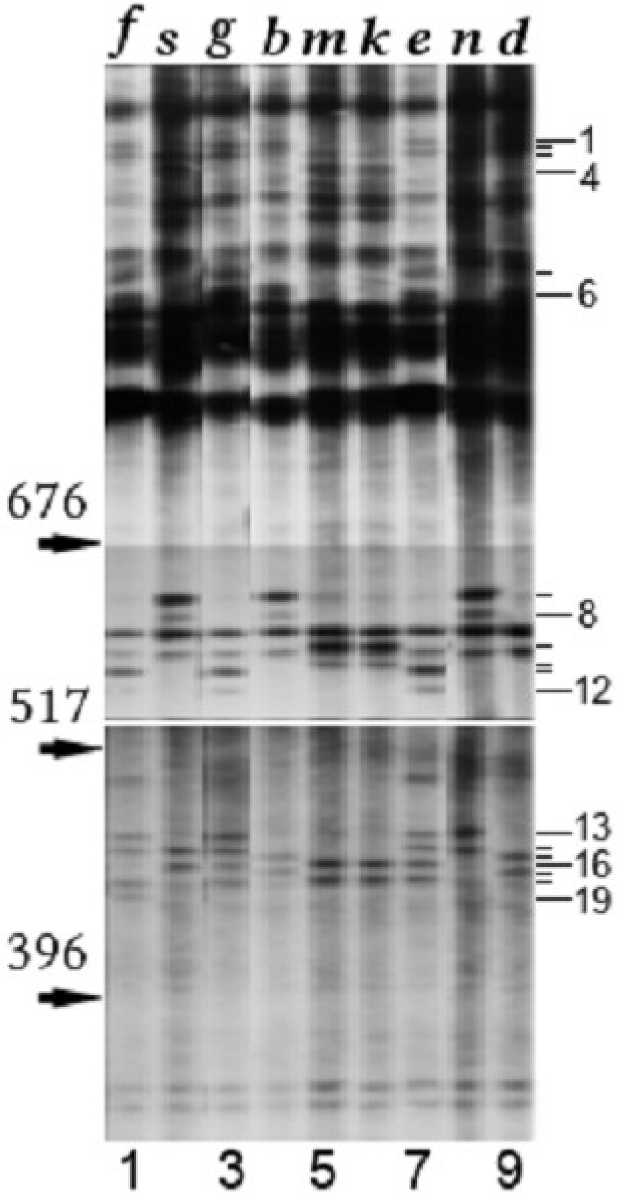

Figure S3

**Cultivar**	**Allele at *Gli-B1***	**Haplotype *GAG56B***	**173**	**182**	**259**	**279**	**348**	**413**	**438**	**474**	**495**	**524**	**532**	**585**	**602**	**694**	**755**
**Insignia**	*i*	1	C	C	G	A	C	C	C	T	A	T	C	A	C	(CAA)18	T
**Pane-247**	*k*	1	C	C	G	A	C	C	C	T	A	T	C	A	C	(CAA)18	T
**Aragon-03**	*o*	1	C	C	G	A	C	C	C	T	A	T	C	A	C	(CAA)18	T
**Gazul**	*r*	1	C	C	G	A	C	C	C	T	A	T	C	A	C	(CAA)18	T
**Intensivnaya**	*n*	2	C	C	T	C	C	C	C	C	A	C	C	A	C	(CAA)8	C
**Spada**	*n*	2	C	C	T	C	C	C	C	C	A	C	C	A	C	(CAA)8	C
**Lesostepka-75**	*q*	2	C	C	T	C	C	C	C	C	A	C	C	A	C	(CAA)8	C
**Salmone**	*s*	2*	no data	T	T	C	C	C	C	C	A	C	C	A	C	(CAA)8	C
**Alcalá**	*b*	3	C	T	T	C	C	C	C	C	A	C	C	A	C	(CAA)7	C
**Anza**	*b*	3	C	T	T	C	C	C	C	C	A	C	C	A	C	(CAA)7	C
**Diego**	*c*	4	C	C	G	C	C	T	C	T	A	C	C	C	C	(CAA)15	C
**Prinqual**	*c*	4	C	C	G	C	C	T	C	T	A	C	C	C	C	(CAA)15	C
**Siete-Cerros-66**	*c*	4	C	C	G	C	C	T	C	T	A	C	C	C	C	(CAA)15	C
**Glenlea**	*e*	4	C	C	G	C	C	T	C	T	A	C	C	C	C	(CAA)15	C
**Marius**	*f*	4	C	C	G	C	C	T	C	T	A	C	C	C	C	(CAA)15	C
**Pernel**	*f*	4	C	C	G	C	C	T	C	T	A	C	C	C	C	(CAA)15	C
**Adalid**	*g*	4	C	C	G	C	C	T	C	T	A	C	C	C	C	(CAA)15	C
**Calodine**	*g*	4	C	C	G	C	C	T	C	T	A	C	C	C	C	(CAA)15	C
**Ardec**	*h*	5	T	C	A	C	C	C	T	T	T	C	T	C	A	(CAA)19	C
**Caia**	*h*	5	T	C	A	C	C	C	T	T	T	C	T	C	A	(CAA)19	C
Pepital	*h*	5	T	C	A	C	C	C	T	T	T	C	T	C	A	(CAA)19	C
**Cajeme-71**	*d*	6	C	C	G	C	C	C	C	T	A	C	C	C	C	(CAA)26	C
**Chopin**	*d*	6	C	C	G	C	C	C	C	T	A	C	C	C	C	(CAA)26	C
**Katepwa**	*d*	6	C	C	G	C	C	C	C	T	A	C	C	C	C	(CAA)26	C
**Chinese-Spring**	*a*	7	C	C	G	C	C	T	C	T	A	C	C	C	C	(CAA)31	C
**Pyrotrix-28**	*m*	8	C	C	G	A	C	C	C	T	A	T	C	A	C	(CAA)20	T
**Etoile-de-Choisy**	*m*	9	C	C	G	A	C	C	C	T	A	T	C	A	C	(CAA)17	T
**Titien**	*m*	9	C	C	G	A	C	C	C	T	A	T	C	A	C	(CAA)17	T
**Astral**	*f*	10	C	C	G	C	G	T	C	T	A	C	C	C	C	(CAA)15	C
**Floreal**	*f*	10	C	C	G	C	G	T	C	T	A	C	C	C	C	(CAA)15	C
**Saratovskaya-39**	*e*	11	C	C	G	C	C	T	C	T	A	C	C	C	C	(CAA)16	C

Figure S4

Figure legends

Figure S1. Schemes of blocks of jointly inherited gliadin bands (APAGE) controlled by alleles at the *Gli-B1* locus studied. ω-gliadins controlled by the *Gli-B5* locus [30] are shown by arrows.

Figure S2. Two-dimensional (APAGE x SDS) gliadin separations of Spanish landraces. a, Barbilla de Carvajales de Alba (*Gli-B1h*); b, Rojo de Humanes (*Gli-B1q*); c, Blanquillo de Toledo (*Gli-B1g*); d, Jeja Candeal (*Gli-B1v*). The long-tailed black arrows indicate γ-gliadins controlled by alleles at the *Gli-B1* locus, the transparent short-tailed arrows and the short-tailed black arrows show the *Gli-A3*-controlled ω-gliadin having a MW of 41 kD ("internal marker") and the *Gli-D1*-encoded γ-gliadins, respectively. The genotype of the Jeja Candeal includes a null-allele at the *Gli-A3* locus ("internal marker" is absent). Only the γ- and ω-zones of the two-dimensional electrophoregram are shown.

Figure S3. The RFLP patterns (restrictase *Taq*I) of some common wheat cultivars carrying different alleles at the *Gli-B1* locus: 1, Chiarano; 2, Salmone; 3, Etruria; 4, Mirtos; 5, Pandas; 6, Pegaso; 7, Santerno; 8, Spada; 9, Tiberio. Alleles at the *Gli-B1* locus are indicated above the lanes.

Figure S4. Nucleotide sequences of the γ-gliadin pseudogene *GAG56B* in the common wheat cultivars studied**.** Positions are counted from the beginning of the primer employed in the DNA amplification**.**

Table S1. Cultivars with different alleles at the *Gli-B1* locus studied by two-dimensional (APAGE x SDS) electrophoresis.

	Cultivar	Country	Allele	Block[1]	Reference
1	Chinese-Spring	China	*Gli-B1a*	Fig. 1o	[6]
2	Alpe	Italy	*Gli-B1b*	Fig. 1h	[53]
3	Anda	Germany			[7]
4	Bezenchukskaya-98	Russia			Unpublished[2]
5	Bezostaya-1	Russia			[26],this work:Fig.2d
6	Courtôt	France			Unpublished
7	Kadett	Sweden			[54]
8	Mironovskaya-808	Ukraine			this work: Fig. 3b, d
9	Perzivan-1	Azerbaijan			[55]
10	Pricama	Italy			[54]
11	Prinqual	France	*Gli-B1c*	Fig. 1i	[54]
12	Siete-Cerros-66	Mexico			[24]
13	Neepawa	Canada	*Gli-B1d*	Fig. 1n	[56]
14	Rusalka	Bulgaria			this work: Fig. 3c, d
15	Kharkovskaya-6	Ukraine	*Gli-B1e*	Fig. 1k	this work: Fig. 3e
16	Saratovskaya-29	Russia			[1]
17	Tselinogradka	Kazakhstan			[7]
18	Candeal de Alcala[3]	Spain	*Gli-B1f*	Fig. 1l	This work[4]
19	Abbodanza	Italy	*Gli-B1g*	Fig. 1j	This work
20	Blanquillo de Toledo	Spain			This work: Fig. S2c
21	Barbilla de Carvajales de Alba	Spain	*Gli-B1h*	Fig. 1m	This work: Fig. S2a
22	Barbilla de Leon	Spain			This work
23	Candeal de Puebla Alemanara	Spain			This work
24	Alpe	Italy	*Gli-B1k*	Fig. 1c	[53]
25	San-Pastore	Italy			[57]
26	Costantino	Italy	*Gli-B1m*	Fig. 1b	[24]
27	Pandas	Italy			[24]
28	Skorospelka-Uluchshennaya	Russia			This work: Fig. 2a, e
29	Hembrilla de Jerga	Spain	*Gli-B1o*	Fig. 1d	This work
30	Milturum-321	Russia	*Gli-B1q*	Fig. 1f	This work

31	Milturum-551	Russia			This work
32	Rojo de Humanes	Spain			This work: Fig. S2b
33	Salmone	Italy	*Gli-B1s*	Fig. 1g	[24]
34	Jeja Candeal	Spain	*Gli-B1v*	new	This kwork: Fig. S2d
35	Negrete de Cañaveras	Spain			This work

Notes

[1]Block of gliadin electrophoretic bands (APAGE) encoded by this allele is shown on the Figures 1 and S1.

[2]Cultivars 2 and 4 were studied by Dardevet, Branlard, Metakovsky (1995)

[3] Samples 18, 20-23, 29, 32, 34, 35 are Spanish landraces

[4]The results of analysis of samples 18, 19, 22, 23, 29-31, 35 are not shown.

REFERENCES for the TABLE S1

[53] Pogna, N.E.; Redaelli, R.; Vaccino, P.; Biancardi, A.M.; Peruffo, A.D.B.; Curioni, A.; Metakovsky, E.; Pagliaricci, S. Production and genetic characterization of near-isogenic lines in the bread-wheat Alpe. *Theor. Appl. Genet*. **1995**, *90*, 650-658.

[54] Jackson, E.A.; Morel, M.H.; Sontag-Strohm, T.; Branlard, G.; Metakovsky, E.; Redaelli, R. Proposal for combining the classification systems of alleles of *Gli-1* and *Glu-3* loci in bread wheat (*Triticum aestivum* L.). *J. Genet. Breed*. **1996**, *50*, 321-336.

[55] Metakovsky, E.; Akhmedov, M.G.; Payne, P.I., A case of spontaneous intergenomic transfer of genetic material containing gliadin genes in bread wheat. *J. Genet. Breed*. **1990**, *44*, 127-132.

[56] Dachkevitch, T.; Redaelli, R.; Biancardi, A.M.; Metakovsky, E.; Pogna,N.E. Genetics of gliadins coded by the group 1 chromosomes in the high-quality bread wheat cultivar Neepawa. *Theor. Appl. Genet*. **1993**, *86*, 389-399.

[57] Pogna, N.E.; Metakovsky, E.; Redaelli, R.; Dachkevitch, T.; Chernakov, V.M. The group 1 chromosomes of wheat contain several loci coding for gliadins. In: Proc 8th Intern. Wheat Genet. Symp. (Beijing, China, July **1993**) eds Z.S.Li & Z.Y.Xin; CASP; 1994, 137-140.
